# Development and validation of a Chinese parental health literacy questionnaire for caregivers of children 0 to 3 years old

**DOI:** 10.1186/s12887-019-1670-9

**Published:** 2019-08-22

**Authors:** Yan Zhang, Mu Li, Hong Jiang, Huijing Shi, Biao Xu, Salla Atkins, Xu Qian

**Affiliations:** 1 Department of Maternal, Child and Adolescent Health, School of Public Health; Global Health Institute, Fudan University, Mailbox 175, No. 138 Yi Xue Yuan Road, Shanghai, 200032 People’s Republic of China; 2 Key Lab of Health Technology Assessment, National Health Commission of the People’s Republic of China, Fudan University, Shanghai, China; 3 Jiading District Center for Disease Control and Prevention, Shanghai, China; 4 School of Public Health, University of Sydney, Sydney, Australia; 5 Department of Epidemiology, School of Public Health, Global Health Institute, Fudan University, Shanghai, China; 6 New Social Research and Faculty of Social Sciences, Tampere University, Tampere, Finland; 7 Karolinska Institute, Stockholm, Sweden

**Keywords:** Young children, Parental health literacy, Anticipatory guidance, Scale development

## Abstract

**Background:**

Given the limited information on parental health literacy measurements, the study aimed to develop and validate the Chinese Parental Health Literacy Questionnaire for caregivers of children 0 to 3 years old.

**Methods:**

We conducted a validity and reliability study of the questionnaire through a cross-sectional survey and test-retest analysis respectively between March and April 2017. We recruited 807 caregivers of children 0 to 3 years old, among them 101 caregivers completed the test-retest assessment with 2 weeks interval. The reliability was determined by internal consistency, spilt-half reliability and test-retest reliability. The construct validity was assessed by confirmatory factor analysis.

**Results:**

The 39-question Chinese Parental Health Literacy Questionnaire was demonstrated high internal consistency (Cronbach’s α = 0.89), spilt-half reliability (Spearman-Brown coefficient = 0.92) and test-retest reliability (Pearson correlation coefficient = 0.82). The confirmatory factor analysis showed that the construct of the questionnaire fitted well with the hypothetical model. The participants’ test scores of the Chinese Parental Health Literacy Questionnaire in the cross-sectional survey were positively associated with caregivers being mothers, more educated, the children with Shanghai *Hukou*, having only one child in the family, and higher family income.

**Conclusion:**

The Chinese Parental Health Literacy Questionnaire demonstrated good reliability and validity, which could potentially be used as an effective evaluation instrument to assess parental health literacy.

**Electronic supplementary material:**

The online version of this article (10.1186/s12887-019-1670-9) contains supplementary material, which is available to authorized users.

## Background

Improving child health is core to the Sustainable Development Goals [1]. In the past decades, the survival rate of children under 5 years old has improved significantly globally. In low- and middle-income countries, however, 250 million children under 5 years old are at risk of not achieving their developmental potential [2]. Early child development largely depends on the quality of nurturing and care provided to the children in the family. Studies have showed that inappropriate caring practice was adversely associated with child development and health [3].

Health literacy is a better predictor of health condition than income, employment, education, race or ethnicity [4]. In China, the 2016 health literacy surveillance reported that only 11.58% Chinese residents had basic health literacy [5]. Caregivers with lower health literacy had difficulty in comprehending important aspects of pediatric anticipatory guidance, including coping with common family emergencies, weighing risks and benefits of routine vaccinations, and conducting home safety checks [6]. Children whose parents had low health literacy often had poor health outcomes, such as poor asthma control and poor glycemic control, especially for younger children [7, 8]. Low health literacy in parents was also associated with a variety of adverse health behaviors, including not practicing breastfeeding [9], poor performance of administering medicine prescribed [10], which could have adverse effects on children’s health.

Currently, there are several scales to assess adult health literacy, such as Test of Functional Health Literacy in Adults (TOFHLA) [8], Rapid Estimate of Adult Literacy in Medicine (REALM) [11] and Newest Vital Sign (NVS) [12]. However, other than the Parental Health Literacy Activities Test (PHLAT) [6], no instrument has been specifically developed for evaluating parental health literacy of caregivers of young children. The PHLAT was designed for parents of children younger than 13 months, and mainly assessing parents’ literacy and numeracy skills in understanding instructions of caring for children [6].

In 2012, the World Health Organization Regional Office for Europe developed a broader and inclusive definition of health literacy, “people’s knowledge, motivation and competences to access, understand, appraise, and apply health information in order to make judgments and take decisions in everyday life concerning health care, disease prevention and health promotion to maintain or improve quality of life during the life course” [4, 13]. This suggests that the measurement of health literacy should be multi-dimensional.

Given the limited information on parental health literacy measurements, our study aimed to develop a Chinese Parental Health Literacy Questionnaire (CPHLQ) for caregivers of children 0 to 3 years old.

## Methods

### Instrument development

**Fig. 1:**
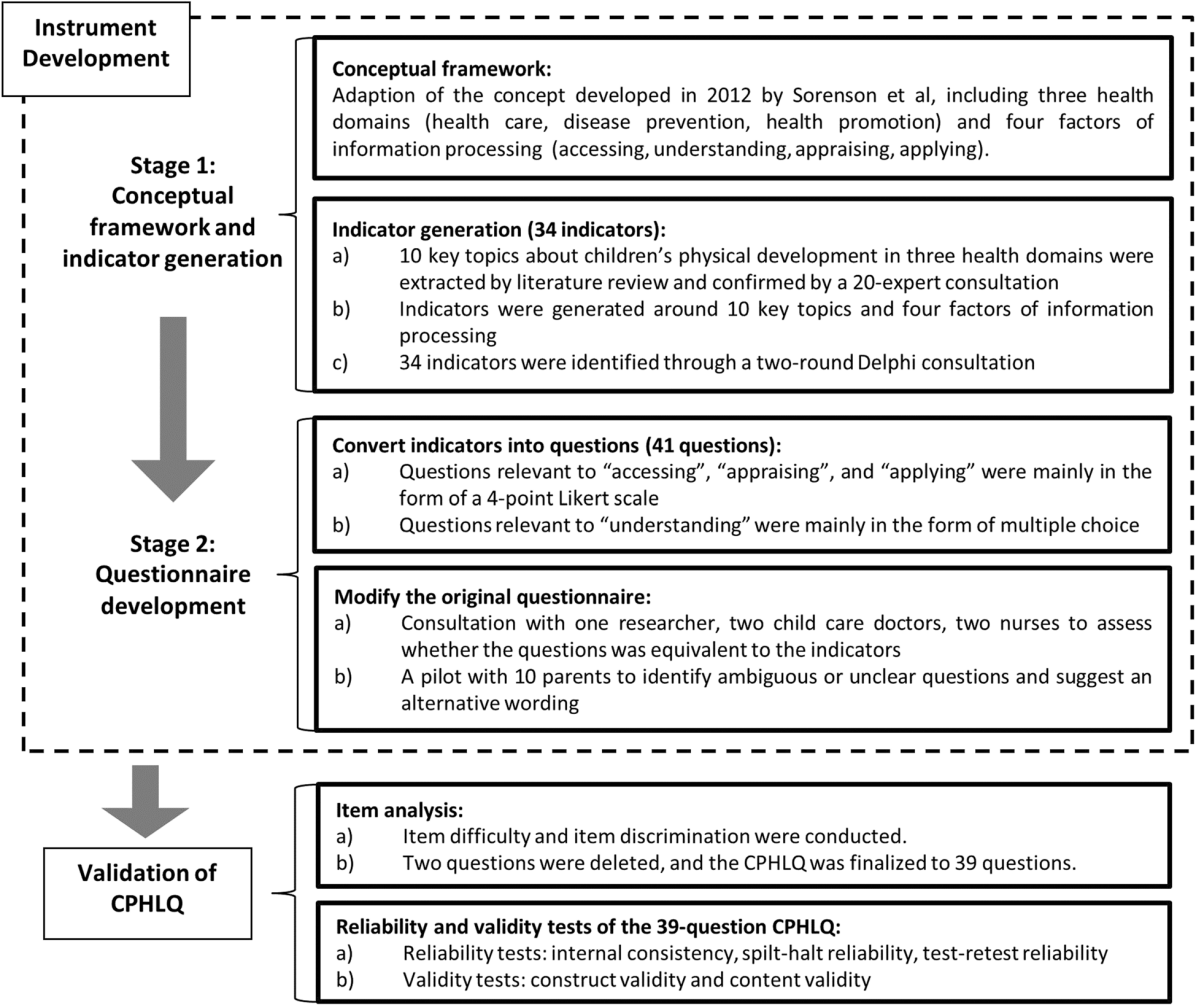
Diagram for the procedures followed to develop the Chinese Parental Health Literacy Questionnaire

The development of the Chinese Parental Health Literacy Questionnaire comprised two stages as illustrated in Fig. 1.

#### Stage 1: conceptual framework and indicators generation

The CPHLQ was based on the conceptual framework developed by Sorenson et al. in 2012, operationalized with a 3 × 4 matrix, including three health domains (health care, disease prevention, and health promotion) and four factors of information processing (accessing, understanding, appraising, and applying) for each domain [13].

Indicators were generated through three steps. Firstly, 10 key topics about children’s physical development in three health domains were extracted from literature review and confirmed by a 20-expert consultation (Table 1). The 20 experts were selected purposively. They are experts in child health care or health education, including researchers, pediatricians and child health care doctors. Pneumonia and diarrhea, the two leading infectious causes of childhood morbidity and mortality, were suggested to represent childhood common diseases in the health care domain. Secondly, several indicators were developed based on the 10 key topics and the four factors of information processing. Thirdly, 14 of the 20 experts completed a two-round Delphi consultation for confirming content representativeness, health literacy relevance, feasibility and significance of these indicators. At the results of these three steps, 34 parental health literacy indicators were identified by consensus [14].

**Table 1:** Key topics about children’s physical development in three health domains

Domain	Key topics
Health care	Pneumonia and diarrhea; antibiotic use; health examination
Disease prevention	Vaccination; obesity and malnutrition; vitamin D and iron deficiency; oral and visual health care
Health promotion	Infant and child feeding; unintentional injury prevention; scientific parental care

#### Stage 2: questionnaire development

Questions were designed based on the 34 indicators. Among them, 29 indicators were directly transformed into 29 questions; for the remaining five indicators, one indicator was converted into two to four questions. As the result, a 41-question CPHLQ were constructed. Each question, reflecting the factors of information processing of “accessing”, “appraising”, or “applying”, was rated with a 4-point Likert scale [15]. Meanwhile, questions relevant to information processing of “understanding” were mainly in the form of true/false questions or multiple choices with four options, designed to test the knowledge level among caregivers. For true/false questions, the correct answer would score 4 points. For multiple choice questions there were 4 options in a question, each option was a true/false question, and one correct choice would score 1 point. Each question also had an option of “Don’t know” which would get a ‘zero’ score. Therefore, each question had a score ranging from 0 to 4. Examples of the questions in the CPHLQ are showed in Table 2.

**Table 2:** Examples of the Chinese Parental Health Literacy Questionnaire

Indicators	Questions
Accessing	
Get information about children’s health checkup	How easy is it for you to get information about your children’s health checkup? ①very difficult ②fairly difficult ③fairly easy ④easy ⑤don’t know
Understanding	
Know about the common manifestations of iron and vitamin D deficiency in children	(1) What are the common symptoms/signs when children have iron deficiency? ①the child looks pale (especially lips, fingernail) ②loss of appetite ③upset ④fatigued ⑤don’t know (2) What are the common symptoms/signs when children have vitamin D deficiency? ①easy to wake up and sweaty at night ②pillow bald patch③muscle weakness ④in serious cases, knock knees and bow legs ⑤don’t know
Understand the harm of dental caries in children	*“If tooth decay occurs in baby teeth, it does not require treatment, because tooth decay will go away after replacing with the permanent teeth.”* Is it true? ①true ②false ③don’t know
Appraising	
Pay attention to children and find the early signs of some common diseases in time	Can you recognize the signs of some common diseases (such as pneumonia, diarrhea) from your child’s physical conditions (such as alertness, body temperature, loose motions)? ①very difficult ②fairly difficult ③fairly easy ④easy ⑤don’t know
Applying	
Ensure children vaccinated according to the local immunization program	Can you always take your baby to scheduled vaccinations as doctor advised? ①always ②in most cases ③sometimes I fail ④rarely do⑤don’t know

The original version of the 41-question CPHLQ was reviewed by one researcher, two child care doctors and two nurses to assess whether the questions were consistent to the indicators. The doctors and nurses came from the department of child health care of a Community Health Center (CHC) in Shanghai, whose main duties were providing medical consultation and health education for caregivers of children. The original version of the questionnaire was piloted with 10 parents to identify any ambiguous or unclear questions and to revise the wording. Minor changes were made to enhance clarity and comprehension.

### Validation of CPHLQ

#### Participants and data collection

The study used a methodological design with a convenience sampling scheme. Usually for a validation study, the recommended sample size for each question is between 2 and 20 subjects; and the total sample of 500 participants is considered as good, 1000 or more as excellent [16]. Eight of the sixteen districts in Shanghai were willing to participate in the study, including three urban districts, three suburban districts and two outer suburban districts. Considering the sample size recommendations and the feasibility, minimum 100 participants from each district (at least 800 participants in total) were required. The target participants were the primary caregivers (including parents, grandparents and other caregivers, like nanny) of children under 3 years old. In Shanghai, the routine child health care is provided by CHCs. Therefore, in each participating district, three CHCs were selected as the study sites, representing high, medium and low social economic status (based on local economic indicators and child health care management rates). A cross-sectional survey was conducted in 24 CHCs from eight districts in Shanghai. Before the survey, two child health care doctors in each selected CHC were invited as co-investigators and were trained about how to recruit participants and complete the self-administered questionnaire.

Caregivers coming to these CHCs between March and April 2017 and meeting the inclusion criteria were invited to join in the survey by the trained doctors. The inclusion criteria were as follows: a) above grade three primary educations, b) able to communicate verbally or literally with the investigators; c) willing to participate in the study. In total 1090 caregivers were approached, and 807 (74.0%) caregivers completed the questionnaire. In order to evaluate test-retest reliability, each study site invited four or five participants to complete the questionnaire again 2 weeks later. Finally, 101 participants completed the questionnaire for the retest. Responses in the first survey by this sample of 101 participants were also used for item analysis.

Data on demographics were also collected from the participants, including caregiver’s relationship with the child, education level, family income, child’s age, gender, and *Hukou* (the Chinese official residency registration by location, which is directly linked to social costs, social benefits and administration). During the survey, the primary spoken language of the study participants was Mandarin, and the questionnaire was administered in Chinese.

#### Item analysis

Based on Classical Test Theory, item analysis was conducted to screen each question’s performance and to ensure the appropriate questions were preserved [17]. The question performance is determined by item difficulty and item discrimination. Item difficulty is calculated as the average score of a particular question divided by the full score of the question, in our study the full score was 4; and for each question the higher this value is the easier the question will be [18]. Item discrimination is examined using the question-total correlation [19]. A question should be deleted, when: a) item difficulty lower than 0.2 or higher than 0.8 [20, 21]; and b) the coefficient of question-total correlation lower than 0.3 [19].

The results were shown in Additional file 1. Based on the above described analysis, three questions were identified to be deleted, “See the doctor in time when suspecting the child has pneumonia”, “Recognize possible risk factors of malnutrition in children”, and “Ensure children fully vaccinated according to the local immunization program”. However, considering the importance of immunization for children, the third question was remained and other two questions were deleted. The 39-question questionnaire across 3 × 4 sub-domains was finalized. The final CPHLQ was organized into three subscales: 12-question for health care health literacy (HC-HL), 16-question for disease prevention health literacy (DP-HL), and 11-question from health promotion health literacy (HP-HL).

#### Reliability and validity tests

Several psychometric properties of the 39-question CPHLQ and the three subscales were assessed.

The internal consistency was measured with Cronbach’s α [22]. Spilt-half reliability was measured with Spearman-Brown coefficient between odd questions and even questions [22]. Test-retest reliability was measured with the Pearson correlation coefficient between the CPHLQ results completed by the 101 caregivers with a two-week interval [22]. In addition, the reliability analysis of the three subscales was also performed. For the whole scale, values greater than 0.70 indicated acceptable reliability [23, 24]. For each of the subscales, values greater than 0.6 were considered as acceptable reliability [25]. The floor or ceiling effects were assessed by the proportion of respondents who received the lowest or the highest score [26].

Given that hypothesized constructs were identified with a priori model, confirmatory factor analysis (CFA) was used to verify the construct validity [27]. The analysis was conducted separately for the three subscales for HC-HL, DP-HL and HP-HL, in which questions were loaded into four factors related to the four information-processing domains of accessing, understanding, appraising and applying. The model fit was considered ‘relatively good’ if the following criteria were met: root mean square error of approximation (RMSEA) lower than 0.08; goodness-of-fit index (GFI) greater than 0.90; adjusted goodness-of-fit index (AGFI) greater than 0.90; comparative fit index (CFI) greater than 0.90; and due to the large sample, χ^2^/df lower than 5 [28, 29]. The content validity was confirmed by the expert panel.

#### Statistical analysis

When calculating the scores for parental health literacy, the weight of each indicator was based on the significance assessed during Delphi consultation, and was equally allocated to the questions related to the indicator. The total score was transformed to percentage grading system, with the full score of 100. The scores of the three subscales and the four competences were also calculated and standardized from 0 to 100. The mean and standard deviation (SD) of CPHLQ scores were calculated. A higher score indicated that the caregiver had higher health literacy. Additionally, descriptive statistics of the participants’ characteristics were tabulated. The relationships between scores and demographic characteristics were assessed with either a t-test or a one-way ANOVA.

CFA was conducted with maximum likelihood estimation by using AMOS 21.0. Internal consistency, spilt-half reliability, test-retest reliability and other parametric tests were computed by using SPSS 20.0. The significance level was set at *P* < 0.05.

## Results

Results of the validation study of the 39-question CPHLQ using a cross-sectional survey are presented below.

### Social and demographic characteristics of participants

In total, 807 caregiver-child pairs participated in the study. There were 551 mothers (68.3%), 178 fathers (22.1%) and 78 grandparents or other caregivers (9.6%). The social and demographic characteristics of the caregivers and their children are shown in Table 3. 64.9% caregivers had college or above education. Among the participants’ children, 52.0% were boys, 67.0% were registered as Shanghai *Hukou*; and 70.5% were the only-child. 70.5% participants reported to have a family monthly income of over RMB 4500 (USD 678) (Table 3).

**Table 3:** Social and demographic characteristics of participants and scores of CPHLQ^a^

	*N* = 807	CPHLQ score (total = 100)	*F* (*P*) or *t* (*P*)
	*n* (%)	Mean (SD)	
Relationship to the child			
Mother	551 (68.3)	74.5 (10.98)	**27.901(<0.001)**
Father	178 (22.1)	69.2 (15.1)	
Grandparents and others	78 (9.6)	68.5 (13.4)	
Caregiver’s education			
Junior school and below	126 (15.6)	64.8 (12.5)	**155.903(<0.001)**
High school	157 (19.5)	67.1 (12.8)	
College	176 (21.8)	74.4 (11.6)	
University or higher	348 (43.1)	77.4 (10.2)	
Child’s age (years)			
≤ 1	522 (64.7)	72.5 (12.6)	0.417 (0.659)
1~2	191 (23.7)	72.8 (12.4)	
2~3	94 (11.6)	73.8 (12.2)	
Child’s gender			
Female	387 (48.0)	73.2 (12.8)	0.870 (0.384)
Male	420 (52.0)	72.4 (12.2)	
Child’s *Hukou*			
Shanghai	541 (67.0)	75.0 (11.7)	**7.362(<0.001)**
Other provinces	267 (33.0)	68.3 (12.5)	
Only-child or not			
Yes	569 (70.5)	73.6 (11.8)	**2.882 (0.004)**
No	238 (29.5)	70.7 (13.8)	
Family monthly income per capita (in RMB)			
<4500	179 (22.1)	69.7 (14.4)	**27.154(<0.001)**
4500~7500	220 (27.3)	73.1 (12.6)	
7500~12,500	183 (22.7)	75.1 (10.1)	
≥ 12,500	156 (19.3)	75.1 (10.8)	
I don’t know	69 (8.6)	68.2 (13.9)	

^a^ *CPHLQ* Chinese Parental Health Literacy Questionnaire

### Reliability

The overall 39-question CPHLQ had high internal consistency (Cronbach’s α = 0.89), high spilt-half reliability (Spearman-Brown coefficient = 0.92) and high test-retest reliability (Pearson correlation coefficient = 0.82). Regarding the three subscales (health care health literacy, disease prevention health literacy, health promotion health literacy), Cronbach’s α coefficient was 0.72, 0.86 and 0.61, respectively; Spearman-Brown coefficient was 0.75, 0.90 and 0.68, respectively; and test-retest reliability coefficient was 0.69, 0.82 and 0.68, respectively.

### Validity

#### Construct validity

The results showed a relatively good fit of all the four-factor structure within the three domains of parental health literacy (Table 4).

**Table 4:** Construct Validity of CPHLQ with goodness-of-fit indices

Model ^a^	Questions	Absolute model fit		Incremental fit		Parsimonious fit
		RMSEA	GFI	AGFI	CFI	χ^2^/df
HC-HL	12	0.05	0.97	0.95	0.94	2.94
DP-HL	16	0.05	0.95	0.93	0.95	3.27
HP-HL	11	0.07	0.96	0.93	0.89	4.87

^a^ Four-factor model of each domain included accessing, understanding, appraising, and applying health information

*HC-HL* health care health literacy; *DP-HL* disease prevention health literacy; *HP-HL* health promotion health literacy; *RMSEA* root mean square error of approximation; *GFI* goodness-of-fit index; *AGFI* adjusted goodness-of-fit index; *CFI* comparative fit index

### Descriptive statistics for the CPHLQ

The mean CPHLQ score of this sample of caregivers of children under 3 years old was 72.8 ± 12.5, ranged 6.0 to 96.8. No floor or ceiling effects was found. The standardized scores of the three subscales (health care, disease prevention and health promotion) were 72.7 ± 11.5, 76.1 ± 16.7, 67.4 ± 14.6, respectively. Furthermore, the standardized scores of the four competences (accessing, understanding, appraising and applying) were 68.7 ± 13.5, 77.0 ± 18.9, 72.6 ± 12.6, 74.3 ± 13.4, respectively.

As shown in Table 3, mothers had higher CPHLQ total scores than fathers and grandparents or other caregivers (*P*<.001). The higher CPHLQ total scores were associated with higher education level (*P*<.001) and higher family income (*P*<.001). In addition, higher CPHLQ total scores were also associated with caregivers’ children had Shanghai *Hukou* (*P*<.001) and were the only child (*P* = 0.004). Scores of the CPHLQ were not significantly associated with child’s age (*P* = 0.659) or gender (*P* = 0.384).

## Discussion

The 39-question CPHLQ was developed for evaluating parental health literacy among caregivers of children 0 to 3 years old in China. The validation study was carried out among primary caregivers who lived in Shanghai. The range of the CPHLQ score is between 0 and 100, a higher score indicates higher parental health literacy level. Psychometric analysis results indicated that the CPHLQ has good reliability and validity, and it could potentially be a useful instrument for assessing parental health literacy for people who care for children aged under 3 years in the Chinese context.

Nutbeam suggested that the measurement of health literacy would be best achieved where content and context were well defined [30]. This study was based on the conceptual framework of health literacy [13], which integrated the content of medical services and public health, and emphasized the individual’s comprehensive literacy abilities, including functional, interactive, and critical health literacy. The application of this conceptual framework provides a better clarity for the connotation of health literacy and provides a theoretical basis for the development of instruments for assessing health literacy. The interactive and critical health literacy involve more advanced cognitive and social skills that can be applied to participate, analyze and better control over life events; while the functional health literacy refers to the basic skills in reading and writing [13]. We found that caregivers scored lower in the competences of accessing, appraising and applying (referring to the interactive and critical health literacy) compared with the competence of understanding (referring to the functional health literacy). This indicates that a comprehensive health literacy intervention is needed to empower caregivers to access, appraise and apply health information. A systematic review showed that a mixed measurement approach can broaden the health literacy concept and enable research to address multiple skills [31]. In the CPHLQ, we used a 4-point Likert scale to determine the ability of “accessing”, “appraising”, and “applying” health information, and used true/false questions or multiple choice to assess the “understanding” of the health information among caregivers.

The psychometric evaluation of the CPHLQ produced plausible results. The overall 39-question questionnaire was reliable, demonstrated by high internal consistency, spilt-half reliability and test-retest reliability (the coefficients were all over 0.8). For the three subscales, all reliability coefficients were over 0.6 which was considered as acceptable reliability for subscales [25]. The results of confirmatory factor analysis showed that the construct of the questionnaire fitted well with the theoretical model. Despite the comparative fit index (CFI) was below the recommended criteria of 0.90 in HP-HL, it still represented a tolerable fit [32, 33]. In addition, we used several methods to ensure the content validity of the questionnaire. We applied the health literacy integration conceptual framework (2012) by Sorenson et al. [13] to construct the CPHLQ. We ensured that the CPHLQ covered the key content of the physical development of children 0 to 3 years old through literature review and expert consultation. We also followed the content development procedures strictly during the questionnaire development process [34] which led to the good content validity.

The study found that mothers’ parental health literacy was significantly higher than fathers, grandparents and other caregivers. This could be due to that in the Chinese culture fathers are less involved in caring for children despite the vital role of fathers in child development [35]. The finding highlighted that in practical terms fathers should not be neglected when carrying out the health education about caring children under 3 years old. In line with other studies, our study found that lower health literacy was significantly associated with lower education level and lower family income [36, 37]. We also found caregivers whose children had Shanghai *Hukou* scored higher than those whose children did not have Shanghai *Hukou*. This is consistent with findings from another study that the level of health literacy among Shanghai residents was higher than the average of the country [38]. This might be partially due to relatively higher education level of Shanghai residents and health care resources, for example health promotion and health information are more accessible among Shanghai registered family [39]. Another interesting finding was that caregivers of two or more children had lower parental health literacy than caregivers of only one child. This indicated that the caregivers of only one child might pay more attention to parenting and child care.

The development and validation of an appropriate instrument is an essential step for parental health literacy research. To our knowledge, this is the first study of developing and evaluating a parental health literacy questionnaire for caregivers of children under 3 years old in China. Using the CPHLQ in a larger and representative sample to determine cutoff point, and in different settings in China are needed. The instrument could potentially be used in other Chinese population, and adapted for the use in other places of the world. Furthermore, the CPHLQ can help to identify the population in need of parenting and child care related information. Therefore, it will be useful for developing targeted interventions to improve the parental health literacy of caregivers of children 0 to 3 years old and the quality of care.

There are several limitations of this study. Firstly, the parenting health literacy presented in this manuscript only involved the physical development and health of children. Secondly, since the participants in this study were all from Shanghai, one of the most developed areas of China, further studies are needed to test the application of instrument in other regions and settings of China. Thirdly, majority questions are based on self-reporting. There might be response bias, for example some participants might overestimate their parenting ability.

## Conclusions

The Chinese Parental Health Literacy Questionnaire has demonstrated good reliability and validity. It could potentially be used as an effective instrument for assessing the Chinese parental health literacy of caregivers of children 0 to 3 years old. The CPHLQ may also help to develop targeted interventions to improve the parental health literacy of caregivers of children under 3 years old and their parenting behaviors.

## Additional file

Additional file 1: Results of item analysis in the pretest and the final version of Chinese Parental Health Literacy Questionnaire. (DOCX 19 kb)

## Data Availability

The raw dataset analyzed in the current study are available from the corresponding author on reasonable request.

## Abbreviations

AGFI: Adjusted goodness-of-fit index
CFA: Confirmatory factor analysis
CFI: Comparative fit index
CHC: Community health center
CPHLQ: Chinese Parental Health Literacy Questionnaire
DP-HL: Disease prevention health literacy
GFI: Goodness-of-fit index
HC-HL: Health care health literacy
HP-HL: Health promotion health literacy
RMSEA: Root mean square error of approximation

## References

[1] UN-DESA (2018). Sustainable Development Goal 3 United Nations, Division for Sustainable Development Goals.

[2] Britto PR, Lye SJ, Proulx K, Yousafzai AK, Matthews SG, Vaivada T (2017). Nurturing care: promoting early childhood development. Lancet..

[3] WHO (2018). Early child development [in Chinese].

[4] Kickbusch I (2013). Health literacy: the solid facts.

[5] National Health Commission of the People’s Republic of China. The level of health literacy of Chinese residents in 2016: Surveillance results. http://www.nhc.gov.cn/xcs/s3582/201711/308468ad910a42e4bbe9583b48dd733a.shtml. Accessed 1 June 2018.

[6] Kumar D, Sanders L, Perrin EM, Lokker N, Patterson B, Gunn V (2010). Parental understanding of infant health information: health literacy, numeracy, and the parental health literacy activities test (PHLAT). Acad Pediatr.

[7] Nakamura D, Ogawa M, Nakamura T, Izawa KP (2018). Impact of Parents' Comprehensive health literacy on BMI in children: a multicenter cross-sectional study in Japan. J Sch Health.

[8] Harrington KF, Zhang B, Magruder T, Bailey WC, Gerald LB (2015). The impact of Parent's health literacy on pediatric asthma outcomes. Pediatr Allergy Immunol Pulmonol.

[9] Kilfoyle KA, Vitko M, O'conor R, Bailey SC (2016). Health literacy and Women's reproductive health: a systematic review. J Women's Health (Larchmt).

[10] Yin HS, Dreyer BP, Foltin G, van Schaick L, Mendelsohn AL (2007). Association of low caregiver health literacy with reported use of nonstandardized dosing instruments and lack of knowledge of weight-based dosing. Ambul Pediatr.

[11] Sanders LM, Thompson VT, Wilkinson JD (2007). Caregiver health literacy and the use of child health services. Pediatrics.

[12] Liechty JM, Saltzman JA, Musaad SM (2015). Health literacy and parent attitudes about weight control for children. Appetite.

[13] Sorensen K, Van den Broucke S, Fullam J, Doyle G, Pelikan J, Slonskaand Z (2012). Health literacy and public health: a systematic review and integration of definitions and models. BMC Public Health.

[14] Zhang Y, Jiang H, Shi HJ (2017). Application of Delphi method to establish an evaluation system of health literacy of parents with children under 6 years old [in Chinese]. Matern Child Health Care Chin.

[15] Sorensen K, Van den Broucke S, Pelikan JM, Fullam J, Doyle G, Slonska Z (2013). Measuring health literacy in populations: illuminating the design and development process of the European health literacy survey questionnaire (HLS-EU-Q). BMC Public Health.

[16] Anthoine E, Moret L, Regnault A, Sebille V, Hardouin JB (2014). Sample size used to validate a scale: a review of publications on newly-developed patient reported outcomes measures. Health Qual Life Outcomes.

[17] Nunnally J, Bernstein I (1994). Psychometric theory.

[18] Kline TJB (2005). Classical test theory: Assumptions, equations, limitations, and item analyses. Psychological testing: A practical approach to design and evaluation.

[19] Hawker GA, Davis AM, French MR, Cibere J, Jordan JM, March L (2008). Development and preliminary psychometric testing of a new OA pain measure--an OARSI/OMERACT initiative. Osteoarthr Cartil.

[20] Lord FM (1952). The relation of the reliability of multiple-choice tests to the distribution of item difficulties. Psychometrika..

[21] Attali Y, Saldivia L, Jackson C, Schuppan F, Wanamaker W (2014). Estimating item difficulty with comparative judgments. ETS Res Rep Ser.

[22] Zhang WT, Dong W (2013). Advanced SPSS statistical analysis [in Chinese].

[23] Sakane Y, Yamaguchi M, Yokoi N, Uchino M, Dogru M, Oishi T (2013). Development and validation of the dry eye-related quality-of-life score questionnaire. JAMA Ophthalmol.

[24] Cronbach LJ (1951). Coefficient alpha and the internal structure of tests. Psychometrika.

[25] George D, Mallery P (2003). SPSS for windows step by step: a simple guide and reference 11.0 update (4th ed.).

[26] Chau PH, Leung AY, Li HL, Sea M, Chan R, Woo J (2015). Development and validation of Chinese health literacy scale for low salt consumption-Hong Kong population (CHLSalt-HK). PLoS One.

[27] Osborne RH, Batterham RW, Elsworth GR, Hawkins M, Buchbinder R (2013). The grounded psychometric development and initial validation of the health literacy questionnaire (HLQ). BMC Public Health.

[28] Bentler PM, Bonett DG (1980). Significance tests and goodness of fit in the analysis of covariance structures. Psychol Bull.

[29] Schumacker R, Lomax R (2004). A beginner's guide to structural equation modeling.

[30] Nutbeam D (2009). Defining and measuring health literacy: what can we learn from literacy studies?. Int J Public Health.

[31] Altin SV, Finke I, Kautz-Freimuth S, Stock S (2014). The evolution of health literacy assessment tools: a systematic review. BMC Public Health.

[32] Kline RB (2011). Principles and practices of structure equation modeling (3rd).

[33] Duong Tuyen V., Aringazina Altyn, Baisunova Gaukhar, Nurjanah, Pham Thuc V., Pham Khue M., Truong Tien Q., Nguyen Kien T., Oo Win Myint, Mohamad Emma, Su Tin Tin, Huang Hsiao-Ling, Sørensen Kristine, Pelikan Jürgen M., Van den Broucke Stephan, Chang Peter Wushou (2017). Measuring health literacy in Asia: Validation of the HLS-EU-Q47 survey tool in six Asian countries. Journal of Epidemiology.

[34] Devellis WR (2016). Scale Development: Theory and Applications [in Chinese].

[35] Friedman D, Masek B, Barreto E, Baer L, Lapey A, Budgeand E (2015). Fathers and asthma care: paternal involvement, beliefs, and management skills. J Pediatr Psychol.

[36] van der Heide I, Rademakers J, Schipper M, Droomers M, Sorensen K, Uiters E (2013). Health literacy of Dutch adults: a cross sectional survey. BMC Public Health.

[37] Sorensen K, Pelikan JM, Rothlin F, Ganahl K, Slonska Z, Doyle G (2015). Health literacy in Europe: comparative results of the European health literacy survey (HLS-EU). Eur J Pub Health.

[38] Ya-fei H, Run-jie C, Xin-feng P, Shen-bing G, Yuan D (2015). Study on healthy literacy among 15-69-year-old residents of Shanghai City in 2012 [in Chinese]. Chin J Health Educ.

[39] Na J, Ji L, Guo-Wei Z, Ke-Li W, Hong-Bo C, Xu Q (2013). Study on the current situation of basic child healthcare service utilization in a central urban area of Shanghai city [in chinses]. Matern Child Health Care Chin.

