# Supplementary material for: Development and validation of a Chinese parental health literacy questionnaire for caregivers of children 0 to 3 years old
Source: BMC Pediatr. 2019 Aug 22;19:293. doi: 10.1186/s12887-019-1670-9 (PMC6704698; doi:10.1186/s12887-019-1670-9)
Supplement: Supplementary file 1 — Results of item analysis in the pretest and the final version of Chinese Parental Health Literacy Questionnaire. (DOCX 19 kb) [file 12887_2019_1670_MOESM1_ESM.docx]

**Additional file 1:** Item analysis and final version of Chinese Parental Health Literacy Questionnaire

| **Domain** | **Question (short description)** | **Difficulty ^a^** | **Discrimination ^b^** | **Screening results** |
| --- | --- | --- | --- | --- |
| **Health Care** | |  |  |  |
| **Accessing** | HC1.1 Get information about diarrhea and pneumonia in children | 0.73 | 0.572** | remain |
|  | HC1.2 Get information about children's health checkup | 0.78 | 0.334** | remain |
| **Understanding** | HC2.1 Understand the basic symptoms of diarrhea in children | 0.78 | 0.650** | remain |
|  | HC2.2 Understand the basic symptoms of pneumonia in children | 0.83 | 0.546** | remain |
|  | HC2.3 Understand the rational use of antibiotics | 0.85 | 0.335** | remain |
|  | HC2.4 Understand the significance of children’s health examination | 0.83 | 0.552** | remain |
| **Appraising** | HC3.1 Pay attention to children and find the early signs of some common diseases in time | 0.75 | 0.417** | remain |
| **Applying** | HC4.1.1 See the doctor in time when children have diarrhea | 0.67 | 0.460** | remain |
|  | HC4.1.2 Use oral rehydration salts prescribed when children have diarrhea | 0.58 | 0.656** | remain |
|  | HC4.2.1 Recognize the possibility of early pneumonia in children by count respirations | 0.56 | 0.547** | remain |
|  | ***HC4.2.2 See the doctor in time when suspecting that children have pneumonia*** | ***0.95*** | ***0.213**** | ***delete*** |
|  | HC4.3 Give children antibiotics prescribed | 0.74 | 0.499** | remain |
|  | HC4.4 Take children to complete the check-up on schedule | 0.87 | 0.344** | remain |
| **Disease Prevention** | |  |  |  |
| **Accessing** | DP1.1 Get information about child vaccination | 0.70 | 0.228* | remain |
|  | DP1.2 Get information about the prevention or management of common childhood health problems | 0.67 | 0.564** | remain |
| **Understanding** | DP2.1 Understands the significance of vaccination to children | 0.87 | 0.499** | remain |
|  | DP2.2.1 Know about the common manifestations of iron deficiency in children | 0.58 | 0.726** | remain |
|  | DP2.2.2 Know about the common manifestations of vitamin D deficiency in children | 0.62 | 0.757** | remain |
|  | DP2.3 Understand the harm of childhood obesity | 0.79 | 0.608** | remain |
|  | DP2.4 Understand the significance of early vision screening in children | 0.88 | 0.437** | remain |
|  | DP2.5 Understand the harm of dental caries in children | 0.84 | 0.551** | remain |
| **Appraising** | ***DP3.1.1 Recognize possible risk factors for malnutrition in children*** | ***0.90*** | ***0.205**** | ***delete*** |
|  | DP3.1.2 Recognize possible risk factors for childhood obesity | 0.89 | 0.666** | remain |
|  | DP3.1.3 Recognize possible risk factors for visual problems in children | 0.73 | 0.575** | remain |
|  | DP3.1.4 Recognize possible risk factors for dental caries in children | 0.93 | 0.565** | remain |
| **Applying** | ***DP4.1 Ensure children vaccinated according to the local immunization program*** | ***0.92*** | ***0.161*** | ***remain*** |
|  | DP4.2 Ensure children have adequate nutrition to prevent nutrient deficiency | 0.82 | 0.676** | remain |
|  | DP4.3 Help children develop good diet and exercise habits | 0.87 | 0.544** | remain |
|  | DP4.4 Pay attention to children's visual development and check regularly | 0.81 | 0.652** | remain |
|  | DP4.5 Help children develop good oral health habits and check regularly | 0.80 | 0.625** | remain |
| **Health Promotion** | |  |  |  |
| **Accessing** | HP1.1 Access to infant and child care related professional information | 0.73 | 0.541** | remain |
|  | HP1.2 Get information about common unintentional injuries in children | 0.63 | 0.565** | remain |
| **Understanding** | HP2.1 Understand the recommendation of breastfeeding | 0.44 | 0.527** | remain |
|  | HP2.2 Know about the feeding strategy of child allergy prevention | 0.74 | 0.559** | remain |
|  | HP2.3.1 Know about precaution of common unintentional injuries | 0.73 | 0.476** | remain |
|  | HP2.3.2 Know about emergence treatment of common unintentional injuries | 0.68 | 0.438** | remain |
| **Appraising** | HP3.1 Identify the reliability of parenting information from different sources | 0.68 | 0.506** | remain |
| **Applying** | HP4.1 Breastfeed exclusively for the first 6 months | 0.76 | 0.384** | remain |
|  | HP4.2 Take appropriate measures to avoid or reduce the occurrence of child allergies | 0.81 | 0.494** | remain |
|  | HP4.3 Choose a professional way to obtain information for the guidance of parenting | 0.83 | 0.385** | remain |
|  | HP4.4 Ensure the safety of the home environment | 0.91 | 0.341** | remain |

^a^ difficulty = the mean score on the question/ the highest score on the question;

^b^: discrimination is examine by using the question-total correlation;

**: *P*＜0.01；*: *P*＜0.05
